# Building a Newborn Screening Information Management System from Theory to Practice

**DOI:** 10.3390/ijns5010009

**Published:** 2019-01-23

**Authors:** Michael Pluscauskas, Matthew Henderson, Jennifer Milburn, Pranesh Chakraborty

**Affiliations:** 1 Newborn Screening Ontario, Department of Pediatrics, Children’s Hospital of Eastern Ontario, Ottawa, ON K1H 8M8, Canada; 2 Department of Pediatrics, University of Ottawa, Ottawa, ON K1H 8L1, Canada

**Keywords:** newborn screening, neonatal screening, laboratory information management system, laboratory information system, LIMS, LIS, screening information management

## Abstract

Information Management Systems are the central process management and communication hub for many newborn screening programs. In late 2014, Newborn Screening Ontario (NSO) undertook an end to end assessment of its information management needs which resulted in a project to develop a flexible IS Information Systems (IS) ecosystem and related process changes. This enabled NSO to better manage its current and future work-flows and communication needs. An idealized vision of a Screening Information Management System (SIMS) was developed that was refined into enterprise and functional architectures. This was followed by the development of technical specifications, user requirements and procurement. In undertaking a holistic full product lifecycle redesign approach, a number of change management challenges were faced by NSO across the entire program. Strong leadership support and full program engagement are key for overall project success. It is anticipated that improvements in program flexibility and the ability to innovate will outweigh the efforts and costs.

## 1. Introduction

Dating back to the early days of computers, a variety of software programs have been utilized to automate numerous laboratory work-flows, processes and related activities. A Screening Information Management System (SIMS) is a set of integrated software components which may or may not be from a single vendor that can be used to automate newborn screening lab work-flows. In addition, a SIMS can be used to manage other aspects of overall laboratory management and programmatic aspects, such as case reporting and follow-up.

Population based newborn screening began appearing in many North American and European countries over 50 years ago. Due to the low number of tests per patient, many programs were initially able to handle their testing and reporting functions using manual work-flows and paper-based lab logs and patient reports. Labs initially employed simple computer functions such as using word processing to create mailing lists/labels and basic reports. Eventually, usage evolved into managing basic lab work-flows and quality metrics using standard software such as spreadsheets and databases.

In the late 1990s and early 2000s, the shift to complex equipment, such as Tandem Mass Spectrometers (MS/MS), that could test for dozens of target diseases and related follow-ups necessitated the development of a more complex information technology infrastructure to manage newborn screening programs. This shift required the development of new, often integrated, software programs to manage the new challenges that were created by these changes. Also, a number of new target disorders required very quick turnaround times (TATs) in order to locate infants who were at risk of early decompensation that could cause severe morbidity or even death. This meant that neonatal screening labs required information systems that could process large batches of results quickly in order to ensure that infants who tested positive for these disorders could be identified in a timely manner.

The current shift into new paradigms for newborn screening, including molecular (DNA-based) screening, is pushing the limits of the original software architectural approaches of these integrated packages. The ability of decision support tools such as CLIR [1] to be linked, potentially in real time via application program interfaces (APIs) [2], also stretches the limits of current SIMS functionality. In addition, the potential of new point of care technologies and the possibility of screening for certain time-critical disorders prenatally have stretched these new paradigms even further. As these needs evolve, a more comprehensive information ecosystem approach to newborn screening SIMS architectures will be required to support the expanding needs of newborn screening systems.

In their paper, “The Ideal Laboratory Information System [3]”, Sepulveda and Young described a set of processes and technology modules that they considered key for the development of an “ideal” comprehensive clinical laboratory information system. An approach to developing an “ideal” SIMS in a newborn screening context is described in this paper. The experiences of Newborn Screening Ontario (NSO) are used to illustrate the various technical and administrative approaches that are involved in undertaking such a project. The potential benefits, impacts and risks of taking this approach are discussed and key lessons are highlighted.

## 2. Theory

### 2.1. Outgrowing NSO’s Current SIMS

NSO is located in the Canadian province of Ontario. Canada has a publicly funded health care system that is managed by its thirteen provinces and territories. Ontario is Canada’s largest province with a population of approximately 14 million people and an annual public healthcare budget of over $CDN 50 billion in 2017. NSO is a fully integrated program that manages all aspects of newborn screening and follow-up activities for children born in the province of Ontario (approximately 140,000 births per year). With the expansion of provincial newborn screening in 2006, the lab and program management was moved from the Provincial public health branch to CHEO, a leading academic paediatric research hospital that is located in the city of Ottawa. NSO receives ongoing annual base funding from the province as well as project-based funding to implement new programs as needed.

**Figure 1:**
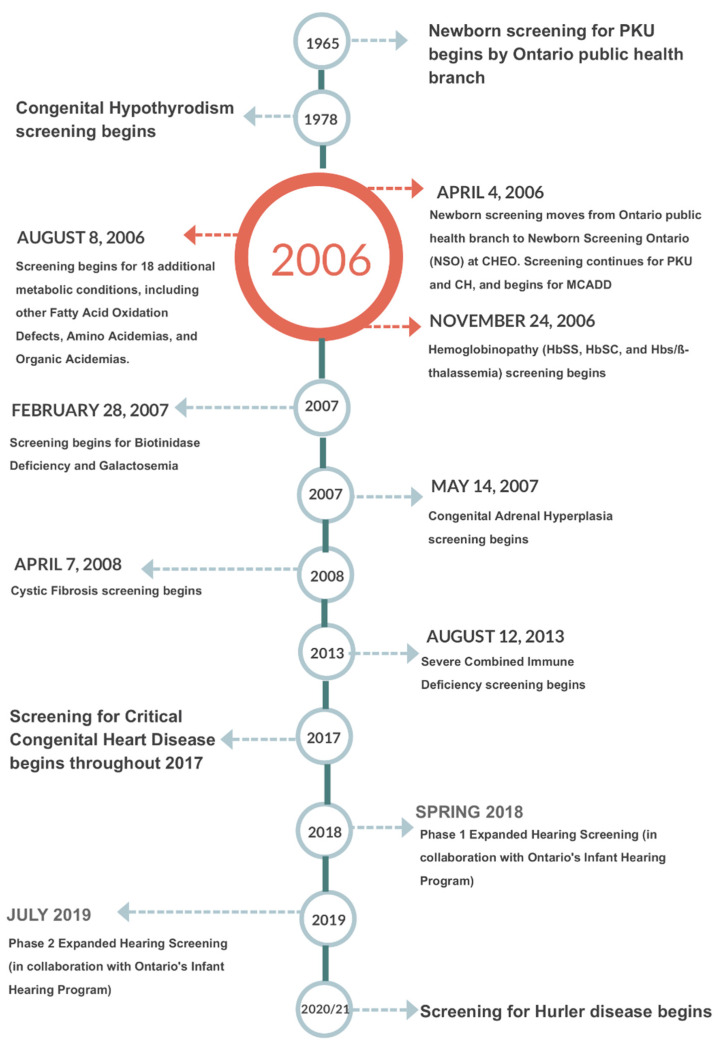
Newborn screening Ontario timeline.

NSO performs blood spot based screening for metabolic and endocrine disorders as well as for hemoglobinpathies, cystic fibrosis (CF) and severe combined immune deficiencies (SCID). Multiple tier testing strategies are also used for a number of these diseases. In addition, NSO offers diagnostic and monitoring testing for many of the aforementioned disorders. Further, NSO coordinates and administers the provincial critical congenital heart disease (CCHD) point of care screening program [4]. NSO is also working with the Ontario’s Infant Hearing Program (IHP) to phase in blood spot testing for hearing loss risk factors including cytomegalovirus (CMV) DNA and certain key genetic risk factors [5]. Figure 1 shows a timeline of newborn screening in Ontario from the beginning of province wide screening for Phenylketonuria (PKU) forward.

Like many other newborn screening programs, NSO coordinates and manages programmatic elements in addition to testing. These include pre-screening education, distribution of collection cards and communications, case management for special screening circumstances such as transfused or premature babies, short term follow-up of screen positive babies and analysis/reporting of key performance indicators. Finally, NSO has an academic mandate and coordinates regular meetings of treatment centre physicians and health care providers, performs research and participates in research collaborations including work that is aimed at understanding the long-term outcomes of screening. It therefore requires an information infrastructure to support these diverse yet closely inter-related set of screening activities.

Newborn screening is a rapidly evolving field. An illustration of the rate of change in the last 15 years is the adoption of the Recommended Universal Screening Panel (RUSP) [6] in the United States. In 2002, the American College of Medical Genetics reviewed 81 conditions and placed 29 of them in a core screening panel, which made up the original RUSP. At that time, the majority of U.S. states screened for only six disorders. Today, all states screen for at least 29 conditions [7]. The RUSP was, and continues to be, influential internationally.

A broader screening panel resulted in more test results per child screened, the need for novel analytical techniques and the need for second/multiple tier testing to improve specificity. Like many other newborn screening labs, the NSO laboratory uses immunoassays, enzyme assays, chromatography, Liquid chromatography–mass spectrometry (LC-MS/MS), Flow Injection Analysis Tandem Mass Spectrometry (FIA-MS/MS), quantitative real-time PCR (qPCR) and primer extension and next generation sequencing techniques for screening. Despite the breadth of techniques used, it is sometimes necessary to reflex first tier screen positive samples to second tier testing methods in order to achieve an acceptable positive predictive value for a positive screening result. An example of tiered testing strategies and complex screening logic is congenital adrenal hyperplasia (CAH). First tier CAH screening relies on the measurement of 17-hydroxyprogesterone. The results are interpreted based on gestational age and birth weight specific screening thresholds. A panel of steroids is measured by LC-MS/MS in first tier positive samples and another set of screening logic is applied to this panel of results.

As the complexity of testing in newborn screening has increased, so has the capacity and expertise in the laboratory. As a result, newborn screening labs are well equipped to provide follow-up diagnostic and monitoring tests for screen positive newborns and those affected by target diseases. In the case of NSO, we offer monitoring for patients with phenylketonuria, tyrosinemia and glutaric aciduria type 1, along with molecular DNA testing for all disease targets of newborn screening. Finally, the pace of change in newborn screening requires that labs continue to develop and implement new screening, monitoring and diagnostic approaches on an ongoing basis. NSO engages in small through large scale research studies that require data to be readily and rapidly available, including access to program, pre-analytical, testing and follow up data.

A single Lab Information System (LIS) solution for overall lab and program management was procured by NSO when it was established in 2006. This system was critical to the initial launch and subsequent development of the program as it provided “tightly-coupled” support for the newborn screening laboratory information flows between equipment, quality Control (QC) and case management. This approach worked well for ensuring the efficient management of lab work-flows’ timely flow of information to follow-up teams, especially in relation to the standard blood spot testing for metabolic disorders. As the complexity of NSO’s mandate increased, it was determined that a broader approach was required to allow for the development of new areas. These areas included the expansion of molecular screening, diagnostic testing, complex screening algorithms, point of care screening, short- and long-term follow-up and sample lifecycle management (see below). As noted above, it was determined that a broader newborn screening information ecosystem needed to be developed.

### 2.2. Designing an “IDEAL” SIMS for NSO

**Figure 2:**
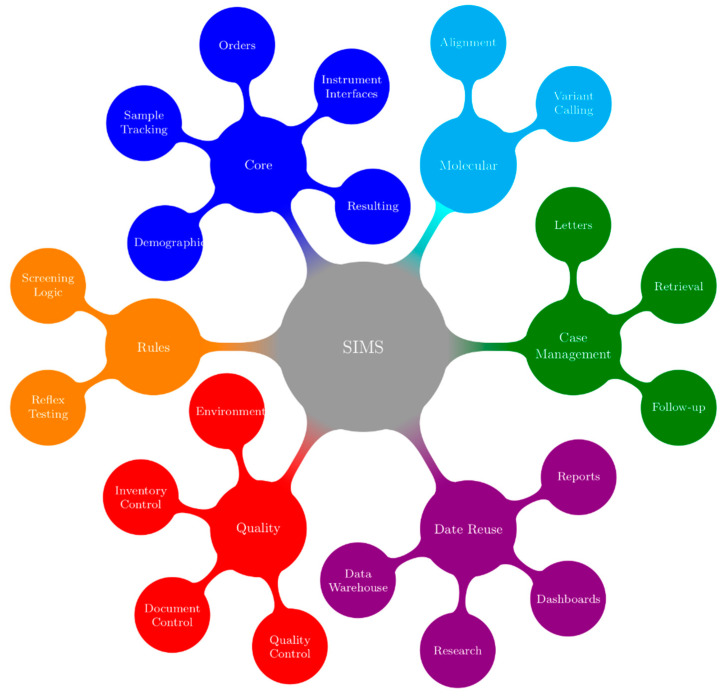
Concepts and Functionality in a SIMS (Screening Information Management System). Each secondary node represents a concept that is addressed by a SIMS. Tertiary nodes show specific examples of SIMS functionally that fall within the linked concept. Colors are used to emphasize the link between concepts and functions.

In their book, McCudden and Henderson noted, “the present and future of health care relies on electronic information” [8]. The complexities that are required to capture complex demographics, manage high sample volumes in a timely manner and integrate multiple test platforms have strained many programs’ abilities to move forward with these innovations. Newborn screening programs require a SIMS that is capable managing all of the data coming into and out of the program. There must also be facilities to customize the SIMS to the needs of the program. The functions of an ideal SIMS for newborn screening programs are outlined in Figure 2.

Screening for CF provides an illustrative example of the requirements of a SIMS; colours in the text are a reference to the concepts/functions that are illustrated in Figure 2. Generally, the first point at which information for a sample enters the SIMS is when samples arrive in the lab and demographics are entered or retrieved from hospital or jurisdictional registries (blue). Samples are punched for analysis (blue) in parallel with demographic entry to reduce turnaround time. The status of all samples from time of receipt in the lab to generation of positive or negative mailers is tracked using real-time dashboards that are located in the lab and administrative areas (purple). In many screening labs, an IRT-DNA CF screening strategy is used, with immunoreactivity trypsinogen (IRT) being used as a first tier biomarker (blue). Testing is performed in accordance with relevant procedures (red). After review and acceptance of quality control results (red), a sample with elevated IRT will generate a request (orange) for CFTR genotyping (cyan). If screened positive for CF (orange), a risk letter will be generated (green). The risk letter incorporates both the IRT (blue) and CF genotype (cyan) results. Many jurisdictions have also implemented or are considering implementing third tier sequencing that will further add to the complexity of this test. The program will refer the screen positive infant to the appropriate care provider and this referral will be documented (green). The NBS program will be notified when the newborn is retrieved and this will be documented (green). Diagnostic testing will be performed and the NBS program will be informed of the final diagnosis (green). Periodically, the program will evaluate CF screening performance. This evaluation requires information from all aspects of the screening program (purple) such as, however not limited to, test turnaround time (blue), time to referral and retrieval (green), final or working diagnosis and positive and negative predictive value (green). Babies and families with CF, CF variants or false positive results may be invited to participate in further program evaluation and research, and the SIMS must facilitate this (green).

While not exhaustive, the CF screen positive scenario provides an overview of the requirements of a SIMS for NBS. Not described above is the need to make changes to the system as disorders, methods, tests and screening logic changes. Once changes are made, an approach to testing the system to ensure that it functions as intended will be required.

In order to realize this vision, NSO launched a large scale project in late 2014 to develop a comprehensive service oriented architecture (SOA) [9] based solution in which information can flow seamlessly through the key areas of the program. The solution consisted of seven key functional modules (services) that combined can achieve this vision, including:

1. Patient Record Management: This module is expected to handle most of the pre-analytical aspects of NSO. The module will be used to receive samples in the laboratory and to enter demographic data for these samples. It consists of a number of processes including, however not limited to: Receiving samples; demographic/clinical indication data entry and validation; test ordering (batch and custom); linking multiple samples to a patient; triggering work-flows for samples that are unsatisfactory for testing and ensuring electronic transmission receipt of key information (e.g., demographics in, results out) via HL7 and related protocols.
2. Laboratory Information System (LIS)/Quality Control (QC): These modules will be expected to handle most of the analytical and QC data including work-flows and dataflows within the NSO laboratory environment.
3. Clinical/medical review: The review and releasing of results is a critical component of laboratory information work-flow between the technologists and medical/scientific staff. This module is designed to apply pre-programmed logic to distill critical lab information in order to produce actionable results in an efficient manner. The Clinical/medical review module will consist of a configurable rules-based system that integrates information from multiple data sources, applies disorder logic and streamlines work-flows to drive decisions. It will also include a rules-based expert system including a web-based graphical user interface (GUI) to support the review and reporting functions, an administrative interface for creating and managing rules and a user configurable knowledge base that is “human readable”. In its first iteration, this functionality will be imbedded in the core SIMS. The possibility of using an independent rules engine service that can be made available to multiple systems in the longer term will also be explored.
4. Case management: This module will be expected to handle most of the post-analytical aspects of NSO including follow up with submitters and treatment centres.
5. Sample Lifecycle Management: The need to track blood collection cards throughout their lifecycle from distribution through transport to storage is a key and sometimes overlooked process within newborn screening program management. A local vendor has worked with NSO to develop a system that helps manage sample card inventory management, track the transport of samples and card usage, monitor expiry dates for filter paper and is working towards tracking in lab samples, off-site storage and destruction.
6. Reporting and Analytics: In order to support the data intensive nature of newborn screening results, NSO has developed a data warehouse to enable user controlled data access and to manage automated and ad-hoc reporting on all types of NSO data.
7. Decision support—Third party products can be linked in to provide decision support to key decision makers to assist in timely, effective decision making.

**Figure 3:**
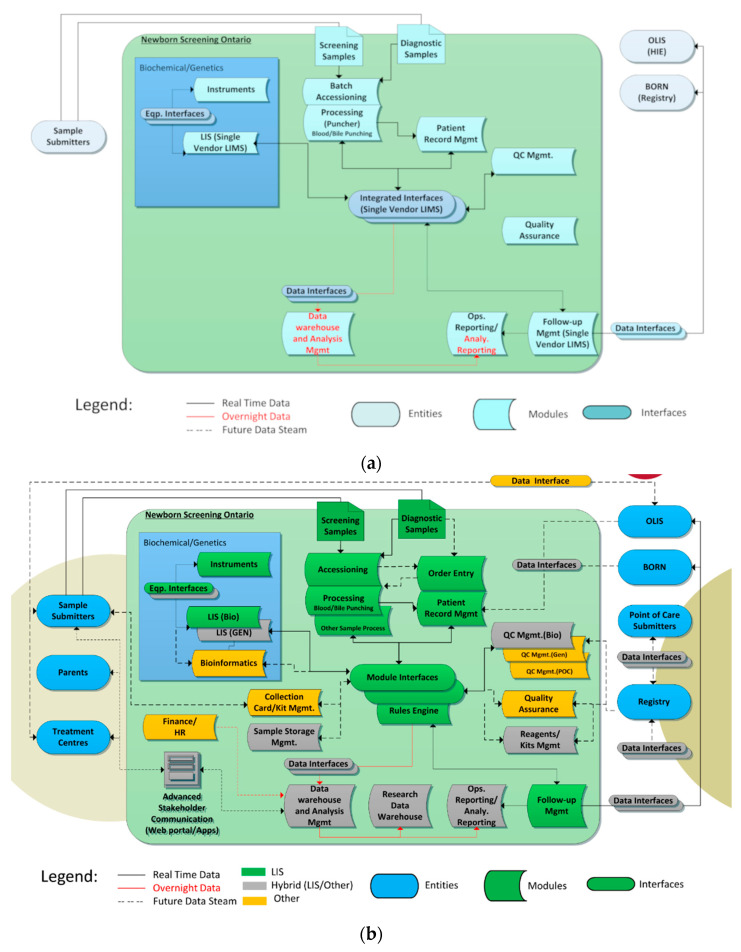
(**a**) NSO Enterprise Architecture—Initial State; (**b**) NSO (Newborn Screening Ontario) Enterprise Architecture—Desired Final State.

These functional modules were combined to produce an enterprise architecture to help guide the project. Figure 3a shows the “tightly coupled” enterprise architecture of the original NSO information system infrastructure and Figure 3b shows the desired final state.

The NSO SIMS components are shown within the large blue box including instruments/interfaces, lab information systems (LISs) and related systems such as data warehouses. The ovals outside the large box show NSO’s data consumers (entities) such as key groups, such as hospitals who submit samples (submitters) and treatment centres who retrieve screen positive and parents. They also show data holding entities such as Health Information Exchanges (HIEs) and data registries. Data-flows are shown via directional arrows.

In Figure 3a, the blue color represents the fact that all functions were handled by a single “tightly coupled” LIS. The new enterprise architecture is more “services” focused with different systems playing different roles. The colors in Figure 3b call out what components are responsible for each function in the new NSO data architecture where green=main LIS (OMNILab), gray = a hybrid of the main LIS and other in house systems and orange = other off the shelf components.

**Figure 4:**
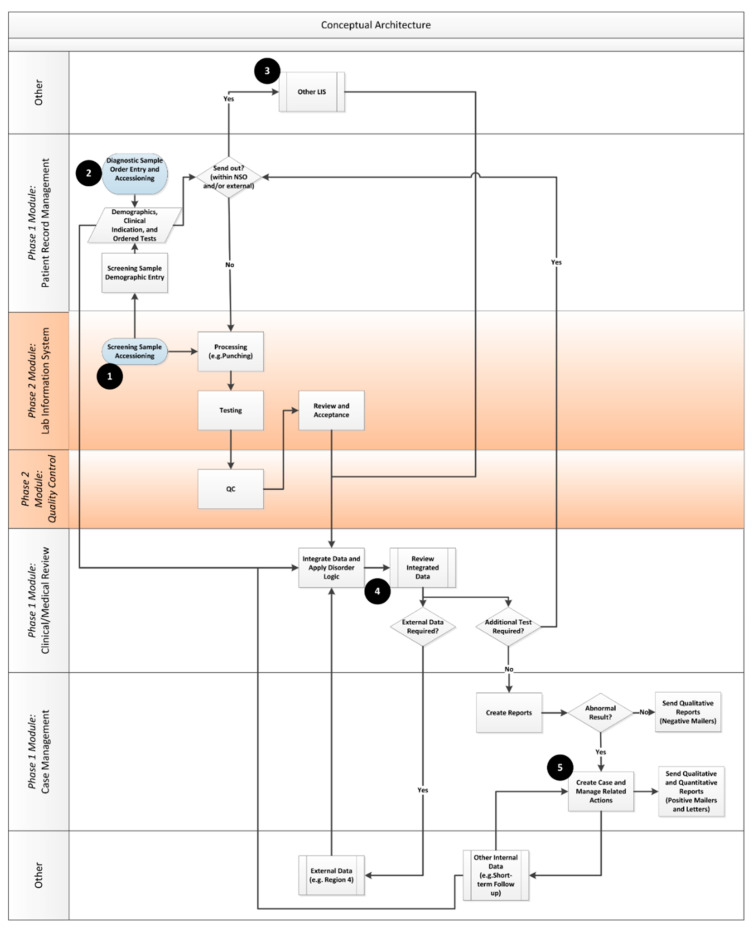
NSO Functional Architecture. The numbers (1–5) provide a reference point to follow the NSO data-flows from entry of the samples into the lab (1) through the sending of reports to submitters (5).

Once the enterprise view of the desired enterprise architecture was developed, the team moved on to ensure that key functional considerations for the system were taken into account by developing a functional architecture. An analogy of the flow from enterprise architecture to a functional one is similar to the link between a building’s architecture and building plans. The enterprise architecture provides a high level idealized view of what can be accomplished across the organization, whereas the functional views (with related user requirements) provide detail about what needs to be built. Figure 4 shows the key conceptual information flows that were developed by the team to guide the project. The modules and phases of implementation are called out on the right side of the diagram. 

## 3. Practice

### 3.1. Technical Considerations

Once the enterprise and functional architectures were solidified, NSO faced a number of choices. An early choice was whether to take a buy, build or hybrid approach to development. When developing any large scale IT infrastructure project, especially one like NSO where there is not a “one size fits all” model available, a decision needs to be made to buy a commercial off the shelf (COTS) solution or to build the software code from scratch. Each of these approaches has pros and cons as discussed below.

COTS systems are supported by a vendor. This can be critically important, especially in areas like newborn screening where access to IT human resources can be severely limited. However, in general, COTS can be less flexible in their ability to meet less common customer requirements. This can lead to the need to either adjust lab work-flows to “conform” to the software or to create manual work arounds that are tracked outside of the system. In addition, requests for system enhancements and/or system fixes generally wait in a queue which, depending on the complexity and urgency of the requirement, can create frustration and potential risks.

Customized software can allow labs to focus on organizational needs and unique user requirements. However, building and supporting software can be very expensive and recruiting and managing qualified software human resources (software engineers, programmers, QA) is not an area of expertise that is associated with laboratory program management. Therefore, undertaking this type of approach exclusively is usually not feasible for most lab programs.

Given the complexity of the architecture involved, NSO chose a hybrid approach using COTS systems wherever possible and then adding in custom solutions to fill in any gaps. Once this choice was made, NSO needed to determine whether to use a single source system or to integrate more than one system to achieve the end goal for its COTS needs. This is often referred to as the choice between integrated single source (one vendor) versus best of breed [10] solutions. Best of breed entails integrating two or more independent solutions that can be from multiple vendors and/or can also be custom built. The main risk of best of breed usually revolves around the integration points. The risk of integration usually resides with the client and can lead to complexity and possibly even conflict if multiple vendors are involved. In addition, a best of breed solution will often require more human resources on the part of the client than working with one vendor.

In order to limit risk and complexity, NSO chose to pursue a best of breed strategy and limit the system to the smallest number of components to meet its needs. Ultimately, the decision of whether to pursue single vendor versus best of breed needs to be based on organizational capacity, budget, risk profile and the maturity of the software options available. That being said, as the needs of the newborn screening community become more complex, the viability of a “one size fits all” model of software delivery will become increasingly more difficult to maintain.

Another set of parameters that were considered was the configurability versus customization of the system. Configurability refers to the ability of trained, usually internal users to adjust key parameters of the software via a user interface. This is in contrast to “customization”, which involves having vendors or custom software developers directly adjust the code of their software to adjust these parameters. A system was chosen that enabled NSO to configure items such as analyte cut-off values, the order of items on a puncher list, the contents of key screen positive results, reports/letters and patient follow-up work-flows.

The more configurable a piece of software, the more individual newborn screening programs can adjust it to meet their specific needs. This can give programs greater control over timelines as they do not have to wait on vendors to customize their software to meet less common end-user needs. As the degree of internal configurability increases, it becomes incumbent on programs to ensure that they have the ability to program and test their configuration changes to ensure that any changes that are made are in fact having the desired effects and no undesirable effects. This approach necessitated a change in human resources at NSO with the creation of new roles for lab subject matter experts to assist the project and the reassignment of lab and follow up personnel to configure the software. Although these HR needs will be higher during the initial build out phase, it is expected that some of the roles will remain in place on a permanent basis once the initial build is over.

The need to balance the ability to test a large volume of time sensitive samples versus enabling a patient-centric view of individual orders was a key driver in determining NSO’s approach. At its core, this dilemma comes down to the ability for a lab system to apply a standard set of tests to a series of samples quickly (known as a batch or sample-centric model) versus the ability to track individuals through the system from accessioning through resulting (known as a patient-centric model). In a screening model where there are hundreds of samples a day, speed is important in order to receive timely results on key sets of time sensitive tests. This need is best served by a sample-centric mode. However, most standard LIS systems are based on a patient-centric model where the details about the individual to be tested are entered into the system and tests and later results are assigned to the patient file.

The majority of requisitions received at NSO are hand filled test requisitions with attached blood filter papers. In order to facilitate timely testing, the filter papers and requisitions are separated and identical pre-printed screening labels are attached to each. These requisitions are sent to data entry clerks to enter the data over the course of the day while the blood spots are immediately punched and tested in the lab. The information is linked in the LIS by the common screening accession number. The issue that can arise is that in “sample-centric mode”, each sample is usually “preassigned” a specific set of tests in order to ensure that they can flow quickly through the lab processes that are needed to produce the standard newborn screening results. The sample-centric system can become particularly unwieldy when there is a need to order different tests on a sample at accessioning (as is the case with diagnostic, monitoring or partial sample panels). To address this, a hybrid set of processes enabling both high volume sample-centric work-flows for standard newborn screening samples while also allowing for patient-centric test ordering was created.

### 3.2. User Considerations

Once architecture and technical considerations had been developed, user needs and requirements were gathered and tracked. A program-wide exercise was carried out to determine desired functionality at a high level, as well as improvements that were necessary to support specific work flows. This “wish list” was documented in order to facilitate the evaluation of overall project success.

Wish list items fell into a number of broad categories, including: The need for better program data management and work-flows; functionality to support/automate processes not currently supported in the LIS that could affect efficiency and safety; the ability to efficiently implement new lab paradigms such as molecular DNA screening and diagnostics; a desire to integrate both diagnostic (patient-centric) and screening (batch-centric) paradigms; the ability to integrate best of breed equipment; case management and follow-up procedure flexibility; and open access to SIMS data for program and laboratory analytics.

Consolidated lists were tracked as part of the project to ensure that the system matched user expectations. Figure S1 User Needs Tracking shows a graphical representation of these consolidated user needs that are categorized by the functional area.

### 3.3. Procurement Considerations

The user needs and functionality statements were used to develop a set of formal business and functional requirements that were utilized in the tendering process. Requirements management software (Enterprise Architect from Sparx Systems) was used to ensure requirement traceability back to the key architecture and functional areas described above. Each business requirement was coupled with a number of related functional requirements. These business requirement (BR) and functional requirements (FR) were used to create an in depth Request for Proposals (RFP) for a SIMS. Table 1 shows an example of a BR and related FRs describing part of the clinical/medical review module that was described earlier in this paper.

The evaluation of the RFP was divided into two phases. The first phase consisted of a written response from qualified vendors that consisted of two sets of criteria. The first criteria was a pass/fail gate that was applied to all mandatory requirements. All vendors that were able to meet these criteria were then scored for non-mandatory requirements. Non-mandatory (desirable) and results were consolidated and averaged. Scoring was weighted based upon an agreed upon relative importance of the requirements. Figure S2 Example of Phase 1 Scoring shows a generic example of phase 1 scoring that was applied for all vendors that met the pass/fail criteria.

The top vendors were then invited to participate for the second phase of the RFP process. In the second phase of the procurement, the vendors were brought in for in depth meetings to describe their proposals and were then asked to provide demonstrations of how various aspects of their systems met NSO’s needs.

Once phase two was complete, NSO used a technique known as Decision Matrix Analysis [11] to determine the best option for moving forward. A Decision Matrix is a table (usually set up in a spreadsheet) where the options you wish to analyze are divided into rows and the factors that influence this decision are divided into columns. The relative importance of these decisions was assigned a weight, usually determined by surveying the key decision makers (e.g., 1 is a low impact and 10 is a high impact). The individual scores for each area were calculated by multiplying the raw score by the assigned impact factor and then the scores were added together to get the final score for each option.

**Figure 5:**
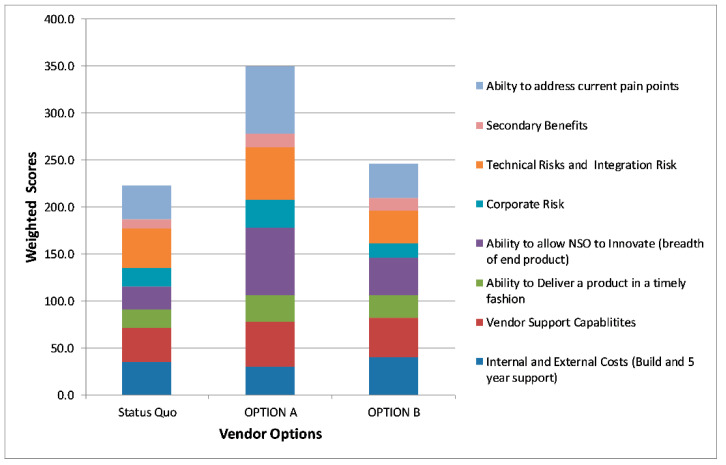
NSO’s Decision Matrix Analysis tool that was used to make final vendor choices.

Eight key factors were identified as critical to the overall decision, including: The internal and external costs including personnel and consultants, software and hardware costs and project costs associated with the build for this option (higher score means lower costs); vendor support capabilities; the ability to deliver a product in a timely fashion; the ability to allow NSO to innovate; technical integration risks including the technical underpinnings of the software and how easy or hard it will be to integrate with other systems (higher score means lower risk); secondary benefits which includes the vendor fixing our current issues without creating new ones. Figure 5 shows an anonymized mock-up of the decision matrix tool that was used to make the final vendor choice.

A COTS solution, OMNI-Lab NBS from Integrated Software Solutions (ISS) Pty Limited, was chosen to fulfill a number of key pieces of the architecture. Following the award of the procurement, ISS and NSO worked on the development of a mutually acceptable contract. Although many non-procurement personnel usually view contract negations as primarily about money, there are in fact a number of other key factors that need to be resolved in addition to the initial and ongoing costs of the software, including the type of contract payment mechanisms such as time and materials versus deliverable based contracts. In the case of NSO, a deliverable based contract was established where contract payments were linked to key milestones. This contractual structure has enabled NSO and the vendor to take a team approach during the design and delivery of the software. Although the upfront effort in defining milestones and working towards mutually agreeable sign offs of project phases can be relatively high, the long term benefits of ensuring that both sides are on the same page during the implementation phase has made the effort worthwhile.

### 3.4. Implementation Considerations

A decision as to how to move forward with system implementation created a challenge as NSO currently has a newborn screening Laboratory Information Management System (LIMS) in place. A staged/agile approach that divided the project into smaller manageable pieces of work, known as sprints, was put in place to mitigate this challenge. The approach enabled the team to focus on key areas of implementation while managing risk. Higher priority areas with lower change management needs were staged earlier than those requiring more change management or where there was currently a system in place to handle day to day operations.

Applying this approach, NSO launched its CCHD screening implementation using the SIMS solution in mid-2017 and blood spot CMV DNA testing of babies not passing their hearing screen was moved into the new system in mid-2018. DBS Screening, which requires the most change management, is currently being deployed. To maximize efficiency, sprints also overlap so that development in one area is started while others are still in progress. This approach has a number of benefits including enabling less risky product acceptance and module configuration across testing and production environments which, in turn, reduces the risk for the more complex deployments.

Several processes to ensure overall build quality have been initiated. The system is deployed across three environments (development, test, production) in order to ensure that all new features can be scoped and configured in the development environment before being tested and finally put into production. This enabled NSO to ensure that new features and configurations were not put into the working environment until they were fully tested and, therefore, do not cause any issues with the production system. Table 2 describes how each environment is utilized in order to ensure full production quality for software, data and configurations.

A number of internal subject matter experts were consulted to develop and deploy test cases for all new system areas in order to ensure that the implications of these changes are fully understood and tested before deployment. All software bugs and requested features are tracked using issue tracking software (JIRA from Atlassian) and, where possible, system testing is automated using a standard testing tool (Ranorex) in order to be able to easily repeat key end to end system tests as new features are deployed. The application of these processes in addition to the staged/agile approach to deployment and implementation is helping to mitigate project deployment risks, especially as the project moves into later and more complex stages.

### 3.5. Organizational and Jurisdictional Considerations

Newborn screening is delivered via a variety of organizational structures around the world and therefore, each program’s overall needs will be different. In spite of this, there are still a number of key areas where specific organizational strategies can be used to ensure the successful delivery of an IS implementation. One critical factor for success is the overall vision and support of senior management. Senior management vision and support has been critical to the overall success of the project to date. Key leadership personnel from NSO, as well as CHEO executive and IS, sit on the project steering committee, which provides guidance and oversight for the project. The project is also represented at the NSO leadership committee in order to ensure that the project aligns with overall NSO strategic direction and goals. Regular progress reports are provided to external stakeholders via the NSO Advisory Council [12].

Ensuring that costs and budgets align with overall program priorities is also key. Many newborn screening programs exist within budget constrained environments where day to day operational priorities take precedent over longer term endeavours such as updating IT systems. Beyond ongoing program support for the IS rebuild, NSO has partially mitigated this issue by treating its IT system as a depreciable asset like other capital equipment and infrastructure and including IT system replacement costs in the overall capital budgeting process. There can also be other novel approaches to working through resourcing issues; for example, other newborn screening programs have begun to pool resources across jurisdictions to share infrastructure in order to keep build and operating costs down.

Another critical area that is often overlooked is the need for strong support from related information technology and communications (ICT) groups that support the newborn screening organization. Often the modern demands of IT can sometimes outstrip the capabilities of some of the older IT systems within a public health setting. In addition, IT and network infrastructures are often procured and managed via different entities or departments than those that are responsible for operating the newborn screening lab and/or program. The emergence of cloud-based technologies will likely help to mitigate some of these pressures in the future as the reliance on related third parties to manage the “nuts and bolts” of infrastructure will likely decrease.

Beyond infrastructure support, jurisdictional considerations can also have a strong influence on a program’s choices of SIMS options. Some jurisdictions are procuring region, state or province wide integrated electronic records solutions, such as those from EPIC Systems Corporation (Verona, WI, USA) [13] and Cerner Corporation (North Kansas, MO, USA) [14]. Newborn screening labs that fall within these jurisdictions are looking to understand whether they can utilize these systems for their SIMS needs and in a few early cases, they have already done so. In addition, some jurisdictions are outsourcing entire newborn screening lab functions, such as MSMS and related biochemistry, along with the related IT requirements. Although these cross-jurisdictional integrated systems can have a definite impact on a newborn screening lab’s SIMS choices, they need not necessarily be a constraint to meeting a lab’s overall needs.

The ability to understand its key architectural and functional requirements can greatly assist in enabling a lab to perform newborn screening functions within an integrated electronic record solution or outsourced service environment. These shared infrastructure solutions may also ease some of the infrastructure pressures and allow labs to focus on their core needs while sharing some of the IS infrastructure costs across multiple functional areas throughout their institutions.

### 3.6. Project Benefits and Impacts

A critical part of any long-term project (or group of projects) is to identify and measure their benefits in order to assess ongoing value. In formal project management terminology, this is often called “benefits realization”. Benefits realization is broadly defined as, “the process of organizing and managing, such that the potential benefits arising from the use of IT are actually realized [15]”. In more practical terms, this can be considered a synthesis of user needs identification as discussed earlier in the paper combined with broader program goals. The project’s key success metrics were determined by the project leadership group at project initiation and are being tracked throughout the project lifecycle. Figure S3 Benefits Realization Tracking shows the benefit realization for key in lab benefits tracking across two different dimensions, namely the impact of the benefit (direction of the arrow) and program area(s) impacted (color of the arrow). In addition, once the SIMS is implemented, NSO will track the impact of the changes on critical clinical program measures such as Positive Predictive Values (PPV) and False Positive Rates (FPRs). Analysis will be undertaken utilizing both the relevant LIS systems and the NSO Data Warehouse. These clinical metrics are reported as part of NSO’s clinical communication to its treatment groups and, more broadly, as part of the NSO annual report. It is expected that given that NSO will be able to achieve more granular control over many of its processes, this will lead to better PPVs and decreased FPRs. Post-launch NSO will continue to monitor these measures in order to ensure continuous quality improvement for its clinical metrics.

## 4. Conclusions

A number of critical lessons in regard to implementing a flexible SIMS architecture were learned through the stages that were described in this paper. One of the key lessons learned was that developing, implementing and deploying a SIMS is about much more than the technology. The SIMS has become the central analytical, process management and communication hub for many newborn screening programs. In order to realize the full benefit of establishing an “ideal” SIMS, it is necessary to engage your full team to ensure that the promise of the new technologies can be achieved. At its most basic level, this requires strong program leadership, vision and strategy. Although the journey can be quite challenging, the benefits of taking a holistic approach to SIMS development in terms of the ability to be flexible and innovate far outweighs the efforts and costs in the long run.

## Figures and Tables

**Table 1:** Business and Functional Requirements for tendering.

Clinical/Medical Review-BR-001: Solution will Receive Input from Multiple Sources. The Solution must Provide the Ability to Receive and Configure Data from Multiple Sources using Standard Messaging Formats (e.g. HL7, XML), Text Files, and Direct Database Connections.				
Related Detailed Requirements				
Name	Description	Mandatory/Desirable	Status—(Mandatory must be 3 or 4)	Description of Functional Solution and Discussion of Status
Clinical/Medical Review-FR-001: Configure input for demographic data	The solution will be able to configure and accept input for demographic data, clinical indications and historical patient information including but not limited to linked samples and previous test results.	Mandatory		
Clinical/Medical Review-FR-002: Configure input for lab results	The system will be able to configure and accept input for laboratory tests and assay/sample results at any stage (e.g. pending, preliminary, post QC, final) from any LIS or directly from instruments. The input must include notes and flags relating to quality control at the sample plate or assay level.	Mandatory
Clinical/Medical Review-FR-003: Configure input for qualitative and quantitative results	The solution will be able to configure and accept input for test results, both quantitative and qualitative ( including but not limited to images such as total ion counts and hemoglobin chromatograms)	Mandatory		
Clinical/Medical Review-FR-004: Configure input for other internal data	The solution will be able to configure and accept input for other internal data such as short term follow-up data including but not limited to diagnostic test results, definitive diagnoses, and courses of treatment.	Mandatory
Clinical/Medical Review-FR-005: Configure input for external data	The system will be able to configure and accept input from external data sources including but not limited to external knowledge bases or analytical results from external entities (e.g. Region 4)	Mandatory		

**Table 2:** NSO Environment Strategy.

Environment Name	Usage	COTS Software Version	NSO Configuration/Rules Version	Data
Development	Developing and testing NSO configuration and rule changes Testing new product builds/product upgrades	Latest software build being tested	Production rules as a base with any changes in progress	Test data only
Test	Deployment testing (DEV to TEST) Sanity testing of new product build and NSO configuration and rule changes Regression testing UAT Training Troubleshooting production issues (e.g., replicating cases on anonymized production data) End-to-end testing with external interfaces with anonymized production data	Same as production For new releases, updated to be same as DEV once DEV build/configuration passed Lancet testing (i.e., production candidate) and close to moving to PROD release During Software builds/product upgrades, contains updated software When no Software product upgrades in progress, same as production	Production rules when testing Software builds / product upgrades and/or troubleshooting production issues New/updated rules when doing final tests on anonymized production data prior to release	Anonymized production data Test data
Production	Production use Sanity testing of new NSO rule changes on production data through the use of test patients	Production	Production	Production data Test data (for test patients only)

## Supplementary Materials

Supplementary materials can be found at http://www.mdpi.com/2409-515X/5/1/9/s1.
