# Supplementary material for: Building a Newborn Screening Information Management System from Theory to Practice
Source: Int J Neonatal Screen. 2019 Jan 23;5(1):9. doi: 10.3390/ijns5010009 (PMC7510236; doi:10.3390/ijns5010009)
Supplement: Supplementary file 1 [file IJNS-05-00009-s001.zip › Figure S2 - Example of phase 1 scoring.pdf]

Evaluation Criteria	Weighting Pass/Fail or out of 10	Pass Fail OR Score (1-4 see above)	Weighted Score (Automatically Calculated)	Comments
**Desirable Functionalities (Scored)**	20%			
**Phase 1**				
**General Business Requirements**	10	3.2	0.8	
**Patient Record Business Requirements**	10	3.2	0.8	
**Case Management Business Requirements**	10	3.5	0.9	
**Clinical Medical Review Requirments**	10	2.5	0.6	
Total Raw Points	40			
Overall Raw Score			3.1	
Section Points	20.0		15.4	
**Corporate Profile/Capability (Scored)**	10%			
Financial and Corporate Viability of the Vendor;	5	1.8	0.3	
Previous experience of the respondent;	10	2.3	0.8	
Quality of references provided by the respondent;	5	2.3	0.4	
Quality of the respondent's proposal	5	2.8	0.5	
Value-adds in the respondent's proposal.	5	2.8	0.5	
Total Raw Points	30		17.5	
Overall Raw Score			2.3	
Section Points	10.0		5.8	
**Technical Evaluation (Scored)**	20%			
Technical Architecture	10	2.5	0.3	
Understanding of Scope	10	2.5	0.3	
System Capabilities	10	2.3	0.3	
Integration capabilities	5	2.3	0.1	
User Interface	5	2.5	0.2	
Fit with existing technology and infrastructure	10	2.5	0.3	
Capability for ongoing changes and enhancements while maintaining current production environment i.e. Test/QA/Production	5	3.0	0.2	
Legacy Data Support	5	3.0	0.2	
Proven design	3	2.8	0.1	
Uptime performance	3	2.5	0.1	
System response time	3	2.5	0.1	
Ability to support or transition to support	3	2.3	0.1	
Data loss prevention/integrity	3	2.0	0.1	
User and system control including: Accountability, access, data integrity controls, auditability and privacy controls	3	2.3	0.1	
Total Raw Points	78		48.5	
Overall Raw Score			2.5	
Section Points	20.0		12.4	
**Implementation and Project Management (Scored)**	20%			
Project Plan	10	2.5	0.4	
Implementation approach and design	10	2.5	0.4	
Proposed team, strength and experience	10	3.0	0.5	
Ability to meet Delivery Schedule	10	2.3	0.3	
Strength of plan	5	2.8	0.2	
Quality control	10	2.8	0.4	
System Verification	5	2.8	0.2	
Enhancement plan	5	2.3	0.2	
Total Raw Points	65			
Overall Raw Score			2.6	
Maximum Section Points	20.0		13.0	
**Training and Technical Support (Scored)**	10%			
Training Plan	5	3.4	0.6	
Support service model	5	2.3	0.4	
Support license agreement	5	2.8	0.5	
Support communication and ticketing	5	2.8	0.5	
Customization service model	10	3.6	1.2	
Total Raw Points	30			
Overall Raw Score			3.1	
Section Points	10.0		7.7	
**Financial Evaluation (Scored)**	20%			
**COMPLETED BY PROCURMENT OFFICER**			18.0	
Total Percentage	100%		72.4	
