# Supplementary material for: Building a Newborn Screening Information Management System from Theory to Practice
Source: Int J Neonatal Screen. 2019 Jan 23;5(1):9. doi: 10.3390/ijns5010009 (PMC7510236; doi:10.3390/ijns5010009)
Supplement: Supplementary file 1 [file IJNS-05-00009-s001.zip › Figure S3 - Benefits Realization Tracking.pdf]

Configurablity

- Manual Workflows Removed
- Control of rules and disease identification
- Control of Workflows
- Greater ability to control configuration

New Equipment and Processes

- Ability to use preferred analyzers
- Ability to implement new lab tests and procedures
- Support all NSO functions (screening, diagnostics, research)
- Ability to handle new paradigms e.g. POC (CCHD), Consenting (IHP)

Program Benefits

- Cost Savings
- Flexibility to undertake on new types of programs
- Case Management flexibility
- Other key functions (financial, card/ kit mgmt, communications)

Data and Reporting

- External Reporting Flexibility (eg. BORN, OLIS, others)
- Access to and understanding of Data (for Data Warehouse etc)
- Native Analytics Capabilities
- Ability to report on key Quality Metrics

Legend:

Arrow direction

- Benefit on track to be achieved
- Unclear whether full benefit will be achieved or not enough data
- Benefit not currently on track to be achieved

Arrow colour (area of impact)

- All of NSO
- Data/Admin function
- Lab function
- Follow-up function
