# Supplementary figures and images for: Building a Newborn Screening Information Management System from Theory to Practice
Source: Int J Neonatal Screen. 2019 Jan 23;5(1):9. doi: 10.3390/ijns5010009 (PMC7510236; doi:10.3390/ijns5010009)

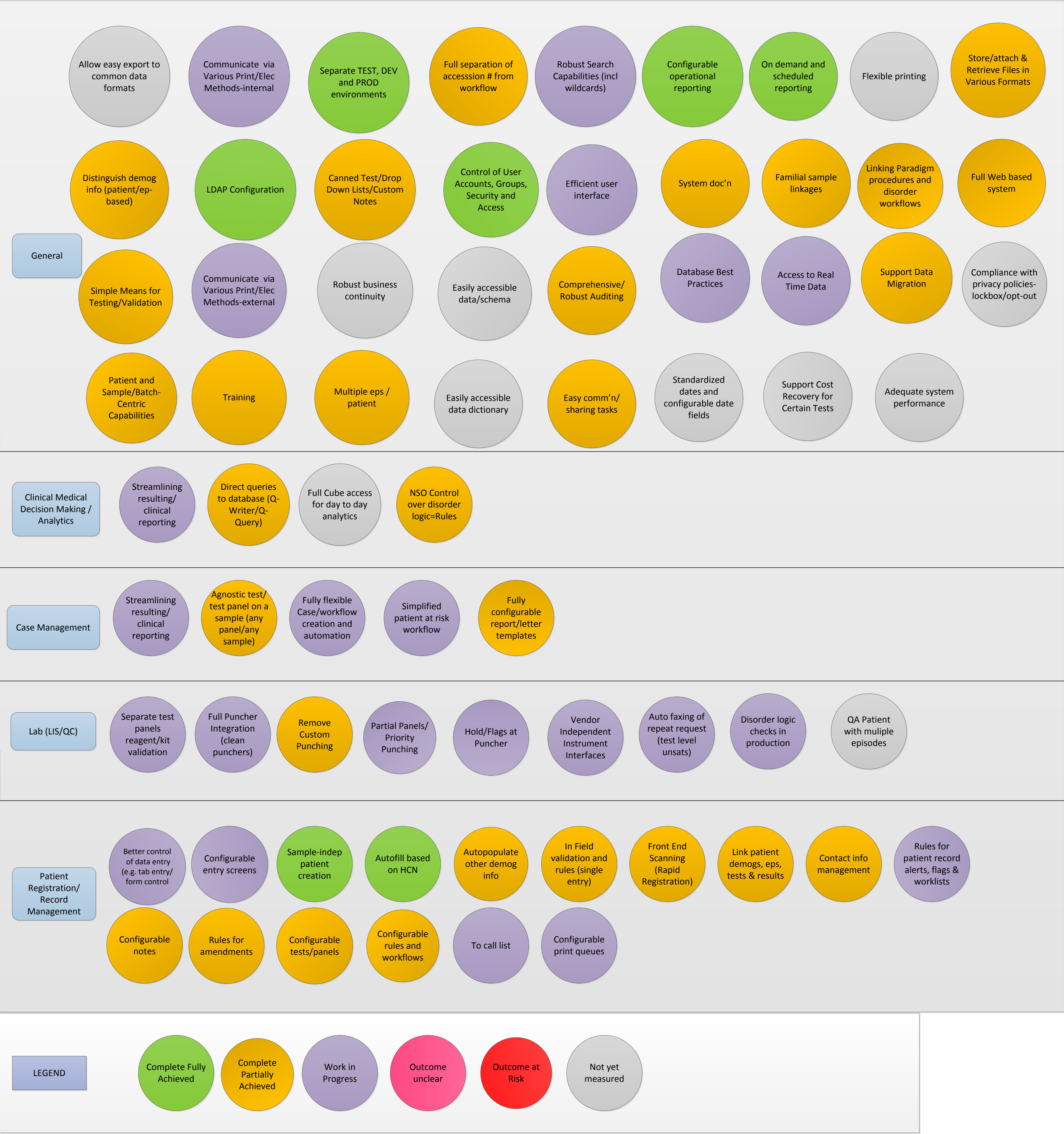

Supplement: Supplementary file 1 [file IJNS-05-00009-s001.zip › Figure S1 - User Needs Tracking.pdf]
